# Isolation and Genomic Sequence of Hepatitis A Virus from Mixed Frozen Berries in Italy

**DOI:** 10.1007/s12560-014-9149-1

**Published:** 2014-05-24

**Authors:** Chiara Chiapponi, Enrico Pavoni, Barbara Bertasi, Laura Baioni, Erika Scaltriti, Edoardo Chiesa, Luca Cianti, Marina Nadia Losio, Stefano Pongolini

**Affiliations:** 1 Sezione Diagnostica di Parma, Istituto Zooprofilattico Sperimentale della Lombardia e dell’Emilia Romagna (IZSLER), 43126 Parma, Italy; 2 Centro di Referenza Nazionale per i Rischi Emergenti in Sicurezza Alimentare, 20133 Milan, Italy; 3 Reparto tecnologia acidi nucleici applicata agli alimenti, Istituto Zooprofilattico Sperimentale della Lombardia e dell’Emilia Romagna (IZSLER), 25124 Brescia, Italy; 4 Servizio Igiene Alimenti e Nutrizione (SIAN), AULSS 4 “Alto Vicentino”, 36016 Thiene (VI), Italy; 5 UFC Sanità Pubblica Veterinaria e Sicurezza Alimentare, Az, Sanitaria di Firenze, 50122 Florence, Italy

**Keywords:** Hepatitis A, Berries, Isolation, Next-generation sequencing

## Abstract

Hepatitis A virus (HAV) was detected in two samples of mixed frozen berries linked to Italian hepatitis A outbreak in April and September 2013. Both viruses were fully sequenced by next-generation sequencing and the genomes clustered with HAV complete genomes of sub-genotype IA with nucleotide identities of 95–97 %.

Hepatitis A cases increased in Northern Italy in the first half of 2013, and all partial viral nucleotide sequences retrieved from the cases belonged to sub-genotype IA and shared identical VP1-2A junction sequence (Genbank accession number KF182323) (ECDC and EFSA 2013). The consumption of berries reported by many of the cases, the reports of previous HAV cases exposed to strawberry and frozen berries, and the identification of mixed frozen berries contaminated with HAV contributed to the hypothesis that the outbreak could be linked to frozen berries (ECDC and EFSA 2013; Rizzo et al. 2013).

In a family cluster in Veneto Region (North-East of Italy), part of the mixed frozen berries eaten 40 days before onset of symptoms was left over, and HAV was detected in those berries by RT-PCR in April 2013 (ECDC and EFSA 2013). Another packet of frozen berries sampled at the house of a HAV case in Tuscany Region in September 2013 was shown positive for HAV by RT-PCR. No apparent relationship was existed between the two episodes.

This study describes the isolation and genomic sequencing of the HAVs detected in these samples of mixed berries and the analysis of their genomes.

HAV has a positive-stranded RNA genome of around 7,500 nucleotides with a single open reading frame (ORF) flanked by 5′ and 3′ untranslated regions (UTRs). The ORF encodes a polyprotein cleaved into four capsid proteins, VP1 to VP4, and nonstructural proteins. A single serotype has been described (Lemon et al. 1992) and, currently, based on the entire VP1 nucleotide sequence (900 nucleotides), six genotypes are recognized, three from humans (I–III), further divided into sub-genotypes A and B, and three of simian origin (IV–VI) (Costa-Mattioli et al. 2003; Desbois et al. 2010). Recently, the analysis of complete genomes evidenced recombination events within the most prevalent sub-genotypes (IA and IIIA) (Belalov et al. 2011) and between sub-genotypes IA and IB (Liu et al. 2010).

The two samples of mixed berries, coded as 112572/2013 and 253042/2013, were tested for HAV by heminested RT-PCR as already reported (Le Guyader et al. 1994), and the positive result was confirmed by sequencing of the amplicon. For virus isolation, samples 112572/2013 and 253042/2013 were inoculated onto Frp/3 (Fetal rhesus monkey kidney, FRhK-4 derivative) cells as described previously (Venuti et al. 1985) for three blind passages. RNA was extracted with RNeasy mini kit (QIAGEN, Milan, Italy) from 230 µl of cell culture supernatant, and HAV presence was examined by RT-PCR (Le Guyader et al. 1994). HAV was isolated only from sample 112572/2013, while sample 253042/2013 showed no infectivity on Frp/3 cells. Next-generation sequencing of the two HAV genomes was performed by amplicon sequencing from the culture-supernatant RNA of the 112572/2013 sample and from the total RNA extracted directly from the 253042/2013 berries sample. CopyDNA was synthesized using HAVRS reverse transcription primer (Tellier et al. 1996). Primer pairs to amplify four overlapping segments of the genome were designed placing primers in conserved regions of HAV full-length genomes available in Genbank. All primers used are listed in Table 1. Four PCR reactions were performed in 25 µl containing 2 µl of cDNA, 0.2 µM of each primer, and 0.5 U of AccuPrimeTM Taq DNA Polymerase High Fidelity (Life Technologies Italia, Monza, Italy) with the following parameters: 94 °C for 30 s followed by 45 cycles at 94 °C for 30 s, 55 °C for 30 s, and 68 °C for 4 min and a final cycle of 68 °C for 8 min. A sequencing library of the purified pooled amplicons was prepared with NEXTERA-XT kit and sequenced on a MiSeq sequencer using a MiSeq Reagent Kit v2 in a 250-cycle paired-end run (Illumina Inc. San Diego, CA, USA). Sequencing reads were de-novo assembled by SeqMan NGen DNASTAR application (version 11.2.1). Two sequences of 7,398 nt and 7,393 nt corresponding to the partial 5′ UTR and to the complete polyprotein coding region of 112572/2013 and 253042/2013, respectively, were obtained with 130 × average depth of sequencing in a single assembled contig for each sample (Genbank Accession number KF773842 and KJ427799). To perform nucleotide alignments, HAV complete genomes having nucleotide identities with isolate 112572/2013 and 253042/2013 higher than 80 % were retrieved from Genbank. Only genomes with specified genotypes were considered. Alignments were performed by ClustalW (Thompson et al. 1994), and gaps were eliminated. Cluster analysis was made on the aligned full genomes (7,186 nucleotides) and VP1 regions (870 nucleotides) with Mega5 (Tamura et al. 2011). Neighbor-joining trees were constructed using the Kimura 2-parameter method (Kimura 1980) with bootstrap test (1,000 replicates). The genomes of both isolates were checked for evidence of recombination with the sequences considered in the alignment by the Recombinant Detection Program (RDP), GENCONV, and BOOTSCAN embedded in RDP3 (Martin et al. 2010). No evidence of intra- or inter-subtype recombination was detected. Genomic cluster analysis showed that 112572/2013 and 253042/2013 HAVs had an overall nucleotide identity of 99.9 %, and that no available complete sequence was highly related to their genomes. The two genomes clustered with other sub-genotype IA sequences with nucleotide identities of 95–97 % (Fig. 1). Based on the VP1 region (Fig. 2), the two Italian sequences clustered with IA viruses with high bootstrap value and, in particular, they had a high nucleotide identity (98.2–99.0 %) with HAV isolates detected in Venezuela in 2005–2006 (Sulbaran et al. 2010). Moreover, the VP1-2A sequences of 112572/2013 and 253042/2013 HAVs had 100 % nucleotide identity with the Italian outbreak sequence KF182323 (from Genbank) indicating their involvement in the outbreak.

**Table 1:** Primer sequences used in this study

Primer	Sequence	Reference
HAVRS	TATTTACTGATAAAAGAAATAAAC	(Tellier et al. 1996)
HAV For4	TACCTCACCGCCGTTTGCCTAGGC	This work
HAV-1969 Rev	ATRTCCATCACTGCACAAGGAGC	
HAV-1000 For	TGATTCATTCTGCAGATTGGCTTACT	This work
HAV-3980 Rev	WTCTCARCTCCATCATTCTAGAGTCC	
HAV For2	GCCGWTGATACTCCTTGGGT	This work
HAV Rev2	CTAGCATCARAAGCAGAGAAATC	
HAV For3	ACGCTTTTTAGAAAGAGTCCMAT	This work
HAV Rev3	ATAAAAGAAATAAACAAACCTCA

**Fig. 1:**
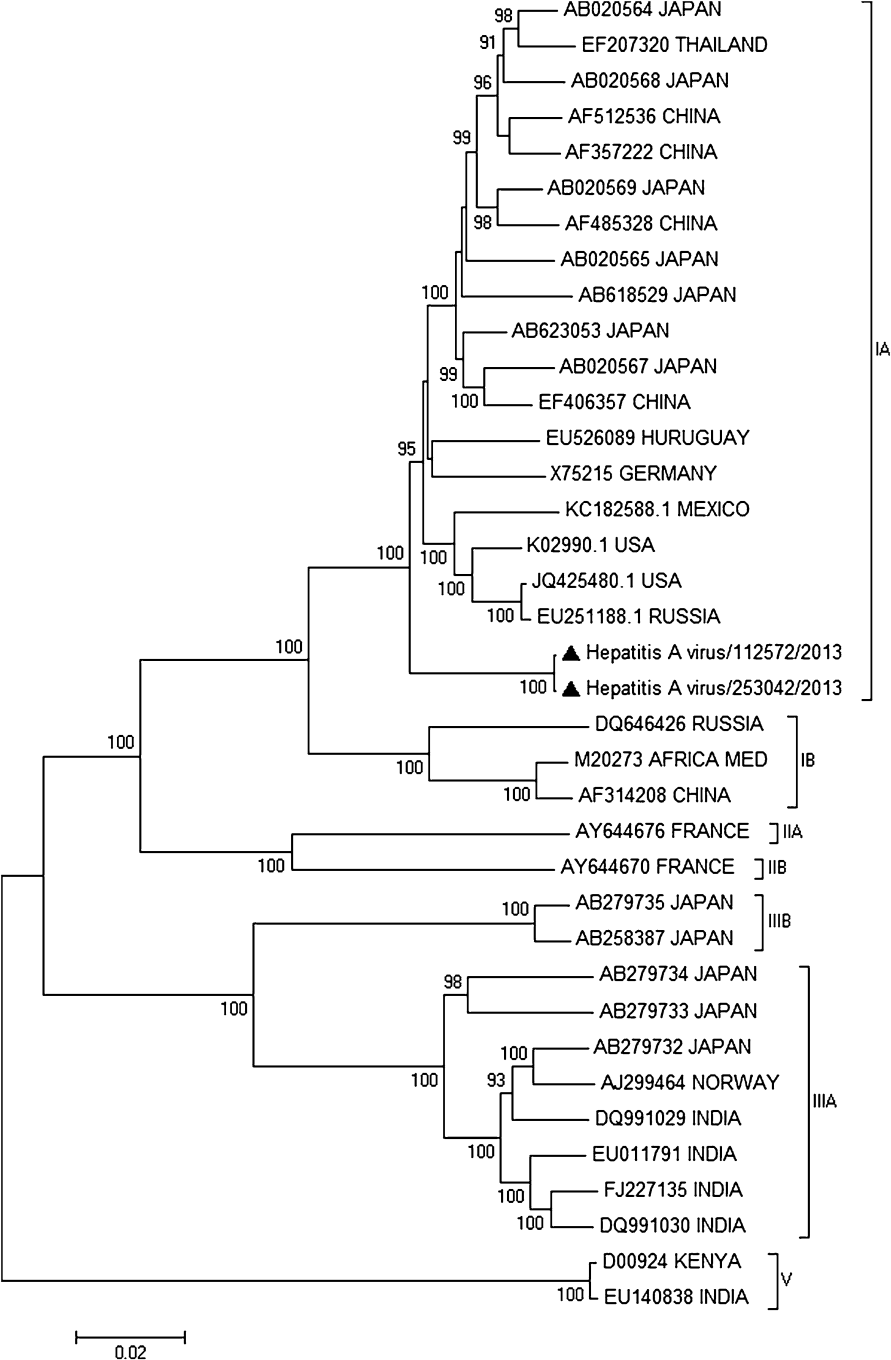
Neighbor-joining tree of the HAV genome. Bootstrap value higher than 90 % is reported

**Fig. 2:**
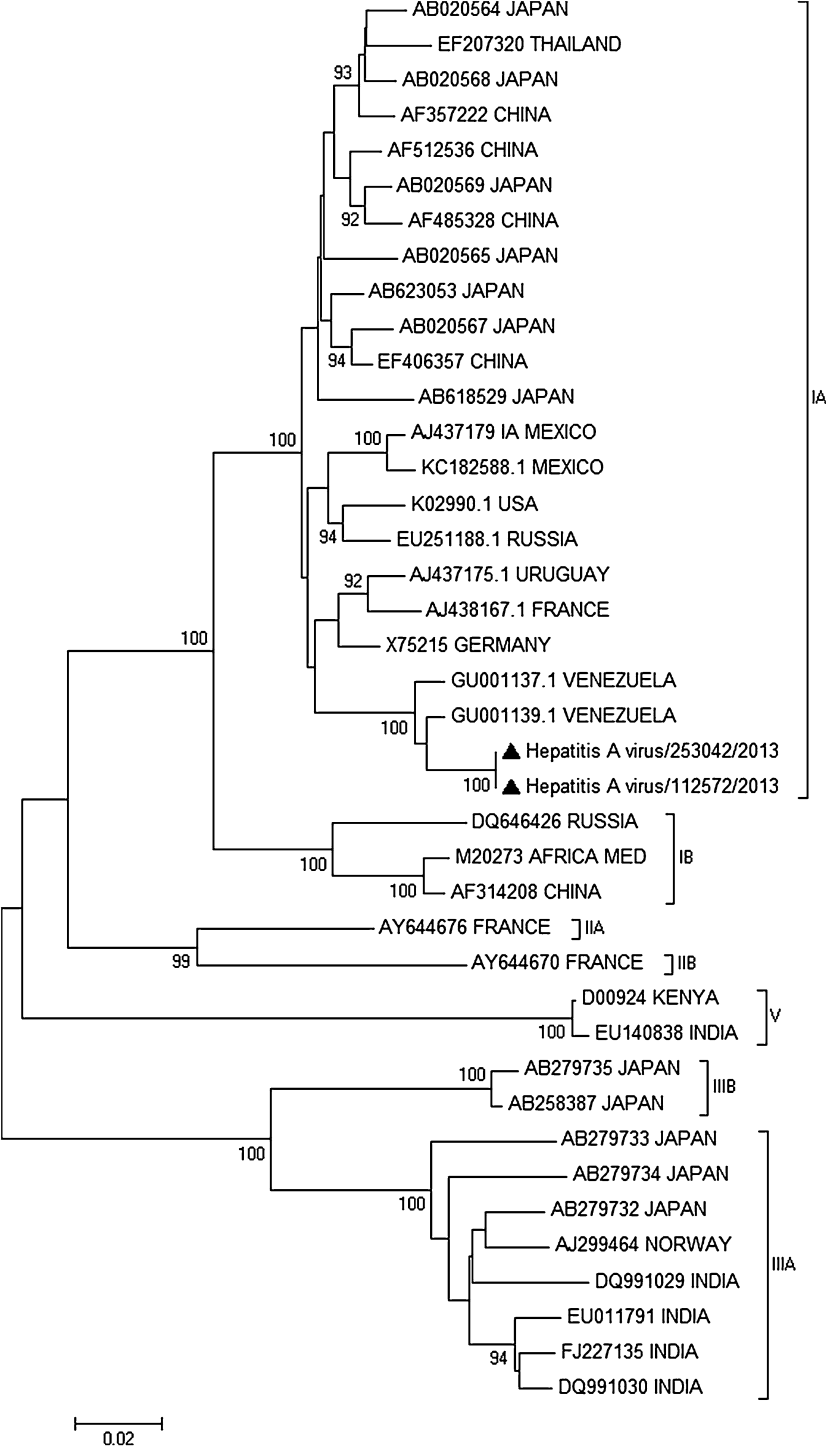
Neighbor-joining tree of the HAV genomic region coding for VP-1. Bootstrap value higher than 90 % is reported

This is the first report of full genomic sequences of sub-genotype IA HAVs from frozen berries linked to the recent European outbreak associated with the consumption of this type of food. The described sequencing methodology proved to be simple, fast and effective both for the culture-isolated virus and for the virus embedded in the food matrix. The sequence of almost the entire genome, besides confirming that these viruses belonged to the IA sub-genotype, allowed the exclusion of recombination events at their origin. While the VP1 sequence indicates high similarity with Venezuelan strains from 2005–2006, the complete genome of the strain described in this study is not clearly related to any of the full genomes of HAV retrieved from the Genbank. Considering that phylogenies produced from sub-genomic regions are generally sufficient to investigate outbreaks over a limited time span but do not reflect unambiguously the epidemiology of HAV over extended periods (Belalov et al. 2011), sequencing of a large number of full-length genomes will contribute to deeper phylogenetic analyses to clarify the origin and chronological pathways of food contamination and disease outbreaks by HAV.
